# Rhodopsin and Melanopsin Contributions to the Early Redilation Phase of the Post-Illumination Pupil Response (PIPR)

**DOI:** 10.1371/journal.pone.0161175

**Published:** 2016-08-22

**Authors:** Prakash Adhikari, Beatrix Feigl, Andrew J. Zele

**Affiliations:** 1 Visual Science and Medical Retina Laboratories, Institute of Health and Biomedical Innovation, Queensland University of Technology (QUT), Brisbane, Queensland, Australia; 2 School of Optometry and Vision Science, Queensland University of Technology (QUT), Brisbane, Queensland, Australia; 3 School of Biomedical Sciences, Queensland University of Technology (QUT), Brisbane, Queensland, Australia; 4 Queensland Eye Institute, Brisbane, Queensland, Australia; National Eye Centre, UNITED STATES

## Abstract

Melanopsin expressing intrinsically photosensitive Retinal Ganglion Cells (ipRGCs) entirely control the post-illumination pupil response (PIPR) from 6 s post-stimulus to the plateau during redilation after light offset. However, the photoreceptor contributions to the early redilation phase of the PIPR (< 6 s post-stimulus) have not been reported. Here, we evaluated the photoreceptor contributions to the early phase PIPR (0.6 s to 5.0 s) by measuring the spectral sensitivity of the criterion PIPR amplitude in response to 1 s light pulses at five narrowband stimulus wavelengths (409, 464, 508, 531 and 592 nm). The retinal irradiance producing a criterion PIPR was normalised to the peak and fitted by either a single photopigment nomogram or the combined melanopsin and rhodopsin spectral nomograms with the +L+M cone photopic luminous efficiency (Vλ) function. We show that the PIPR spectral sensitivity at times ≥ 1.7 s after light offset is best described by the melanopsin nomogram. At times < 1.7 s, the peak PIPR sensitivity shifts to longer wavelengths (range: 482 to 498 nm) and is best described by the combined photoreceptor nomogram, with major contributions from melanopsin and rhodopsin. This first report of melanopsin and rhodopsin contributions to the early phase PIPR is in line with the electrophysiological findings of ipRGC and rod signalling after the cessation of light stimuli and provides a cut-off time for isolating photoreceptor specific function in healthy and diseased eyes.

## Introduction

In macaques, the pupil light reflex (PLR) measured during continuous light exposure and post light offset after pharmacological blockade of outer retinal rod and cone photoreceptors shows a sustained constriction that closely matches the spectral sensitivity of inner retinal melanopsin expressing intrinsically photosensitive Retinal Ganglion Cells (ipRGCs) (peak ~482 nm) [1]. In humans, the spectral sensitivity of the post-illumination pupil response (PIPR) measured at the plateau of the sustained constriction [1,2] and at a single time 6-second after light offset [3], is entirely described by the melanopsin spectral nomogram. Together these measurements confirm that when the PLR is measured in the dark (i.e. no light adapting background field), the PIPR quantified using the plateau and/or 6 s PIPR metrics represents the activity of the melanopsin photopigment alone.

The PIPR has been measured in many studies using a high irradiance, short wavelength stimulus light near the melanopsin peak spectral response to directly assess melanopsin function as a biomarker for retinal disease (for review; Feigl and Zele [2]). Researchers evaluating the PIPR have used different metrics for analysis [1,4–15], with the plateau and the 6 s PIPR metrics showing the least variability [3]. The rationale for quantifying the PIPR at 6 s was that it identified the largest net PIPR amplitude difference between the PIPR in response to short (467 nm) and long (640 nm) wavelength lights [6]. No prior study evaluated photoreceptor contributions to the early redilation phase of the PIPR before 6 s post-stimulus to determine the first post-stimulus time when melanopsin completely mediates the PIPR. Outer retinal rod and cone photoreceptors provide extrinsic inputs to ipRGCs [16,17], however it is not known if the PIPR receives such extrinsic inputs in the early pupil redilation phase after light offset. Here, we evaluate the spectral sensitivity of the PIPR at a range of times after light offset to determine the inner and outer retinal photoreceptor contributions to the early redilation phase of the PIPR. We use spectral sensitivity measurements to determine the first time after light offset when the PIPR is solely controlled by melanopsin input to ipRGCs.

## Methods

### Participants and Ethics Statement

The data presented in this study are the results from a new analysis of previously recorded pupil traces [3,18]. The PIPR spectral sensitivity was derived from the pupil traces of two participants (32 year old female, 31 year old male) with no ocular pathology and not under any prescription medication known to affect the pupil light reflex. They had normal visual acuity, colour vision, visual fields, and no lenticular opacities (Grade 0, Lens Opacities Classification System, LOCS III, Chylack et al. [19]). The PIPR was measured between 10 AM and 5 PM to limit any attenuation of the PIPR amplitude that occurs in the evening nearer to the time of melatonin onset [8,10]. To minimise fatigue and sleepiness, each participant was tested for ≤ 1.5 hours per day and each participated for approximately 15 hours in total.

While the variability of all current PIPR metrics has been evaluated [3], there is no report on the variability of the PIPR between light offset and 6 s post-stimulus. To determine the amplitude and intra- and inter-individual coefficient of variation (CV) of the PIPR at various times between light offset and 6 s post-stimulus, a new analysis was conducted on the pupil data from 20 healthy participants (age; 57.1 ± 10.7 years (mean ± SD), range: 35–74 years) collected for a different study using the same instrumentation [18]. These 20 participants were not involved in the spectral sensitivity experiments but served to provide data on variability. The participants met the inclusion criteria outlined above; 19 participants had no lenticular opacities (Grade 0, LOCS III) and one had a Grade 0.5 cataract that did not affect the PIPR amplitude. There was no effect of age on the PIPR amplitude in the participants (Linear regression; r^2^ = 0.005, F_1,18_ = 0.09, p = 0.77) in agreement with literature [4,20].

All experimental protocols were approved by the Queensland University of Technology Human Research Ethics Committee (approval numbers: 080000546 and 1400000793) and conducted in accordance with the tenets of the Declaration of Helsinki. Written informed consent was obtained from all participants after explaining the nature of the experiment.

### Pupillometer

**Fig 1:**
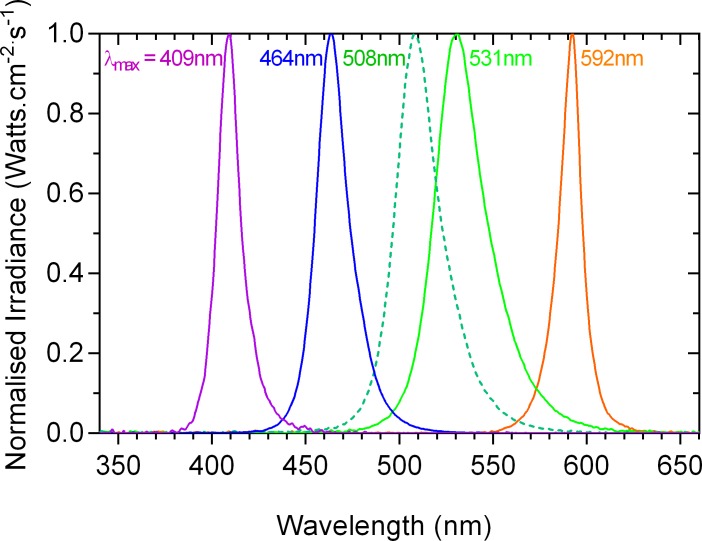
Spectral output of the primary lights. The bandwidths at half maximum output (in nm) are specified in parentheses after the dominant wavelengths (in nm) of the primary lights: 409 (14), 464 (20), 508 (27), 531 (31) and 592 nm (14 nm).

The PIPR was measured using a custom-designed Maxwellian view pupillometer [2,4,21] which comprised five primary lights generated using narrowband LED sources (Fig 1) imaged in the pupil plane of the left eye via two Fresnel lenses (100 mm diameter, 127 mm and 70 mm focal lengths; Edmund Optics, Singapore) and a 5° light shaping diffuser (Physical Optics Corp., Torrance, CA, USA) to provide a 41° diameter light stimulus (retinal image diameter: 17.9 mm). The consensual PIPR of the fellow right eye was recorded under infrared LED illumination (λ_max_ = 851 nm) with a PixeLINK camera (IEEE^-1^394, PL-B741 FireWire; 640 x 480 pixels; 60 frames/s; PIXELINK, Ottawa, ON, Canada) through a telecentric lens (2/3-inch 55 mm and 2 X extender C-Mount; Computar, Singapore). Custom Matlab software (version 7.12.0; The Mathworks, Inc., Natick, MA, USA) was used for operating the stimulus presentation, pupil recording and analysis. A Spectroradiometer (StellarNet, Tampa, FL, USA) measured the LED spectral outputs (Fig 1) and a calibrated ILT1700 Research Radiometer (International Light Technologies, Inc., Peabody, MA, USA) measured the light output in radiometric units (Watts.cm^-2^.s^-1^ and converted to log quanta.cm^-2^.s^-1^ [22]).

### Pupillometry and Spectral Sensitivity Measurements

Spectral sensitivity of the PIPR between 0.6 and 5.0 s after light offset was measured by determining a criterion PIPR amplitude at each test wavelength. This period was chosen to account for the time (0.6 ± 0.3 s) after light offset for the pupil to reach the peak constriction amplitude in response to the 1 s stimulus pulse [3] and on the grounds that the PIPR is already known to follow the melanopsin spectral nomogram for times ≥ 6 s [1–3]. To optimise the use of criterion pupil light reflex, Webster et al. [23] recommended the selection of large stimulus fields, regions of steep slope on the pupil response versus retinal irradiance curves, and observers with the steepest slopes and lowest noise. Accordingly, we measured the criterion PIPR with a 41° stimulus on two participants who showed the lowest variability among the five participants from our previous study and by using the regions of steep slope (above melanopsin threshold ~11.0 log quanta.cm^-2^.s^-1^ retinal irradiance) from the 6 s PIPR amplitude versus retinal irradiance data (Figure 6 in Adhikari et al. [3]).

The range of irradiances required to produce a criterion PIPR was estimated using the 6 s PIPR amplitude versus retinal irradiance curves from Adhikari et al. [3] and the plateau PIPR spectral sensitivity data from Gamlin et al. [1] and Markwell et al. [5]. The pupil traces used to estimate the 6 s PIPR criterion can be used to evaluate the spectral sensitivity of the PIPR measured at other post-stimulus time periods (plateau, AUC early and late) and can therefore also be used for measuring the PIPR spectral sensitivity between light offset and 5.0 s post-stimulus. The irradiances at each wavelength were altered to achieve a criterion PIPR amplitude. The stimulus retinal irradiance at the criterion PIPR amplitude depended on stimulus wavelength and ranged from 13.0 to 15.3 log quanta.cm^-2^.s^-1^. The criterion PIPR amplitude depended on the time after light offset, with the amplitude decreasing with increasing post-stimulus time. The criterion PIPR was always > 15% of baseline pupil diameter (average of 10 s pre-stimulus pupil diameter in the dark) at all selected times, taking into consideration that the intra-individual coefficient of variation (CV) of the 6 s PIPR is about 10% [3].

**Fig 2:**
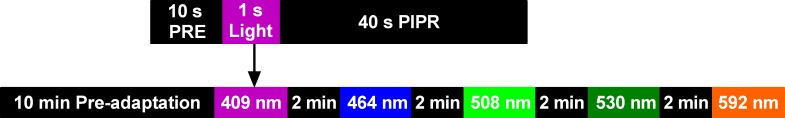
Schematic of the pupillometry protocol. Each experimental session started with 10 minutes pre-adaptation. The order of presentation of the stimulus wavelengths was randomised to maintain a minimum difference of 100 nm between successive stimuli. The example test protocol for the 409 nm stimulus (upper schematic) was common for all wavelengths. There was a two-minute inter-stimulus interval between the tests to allow for the pupil to return to the baseline size. A minimum of four irradiances were presented in 0.2 log quanta.cm^-2^.s^-1^ intervals at each wavelength and a minimum of three repeated measurements were recorded at each irradiance. PRE = pre-stimulus; PIPR = post-illumination pupil response.

To eliminate the influence of prior light exposure on the PLR, the participants were adapted to the room illumination (0.0003 lux) for 10 minutes before all experimental sessions [3] (Fig 2). The left pupil was dilated (1% Tropicamide) to maintain a constant retinal irradiance during light stimulation [24]. A 1 s light pulse was used because it produces larger PIPR amplitudes compared to longer stimulus durations (10 s and 30 s) [3,6,10]. To control for the effect of proposed melanopsin bistability, the difference in the wavelength of successive test stimuli was always greater than 100 nm.

Retinal irradiances were estimated using the model of van de Kraats and van Norren [25] using the corneal irradiances of the lights and correcting for pre-receptoral filtering (not including macular pigment). Pre-receptoral filtering by macular pigment was not taken into account because the macular region (up to 2 mm eccentricity) in humans is devoid of ipRGCs [16,26] and the macular pigment optical density is negligible at 10° eccentricity [27]. Therefore, any effect of macular pigment density will not be significant for the large field size (41° diameter). The retinal irradiances required at each wavelength to produce the criterion PIPR at different PIPR times were normalised to the peak and described by a best-fitting vitamin A_1_ photopigment spectral nomogram [28] with a peak in the range of opn4 (melanopsin) photopigment [16]. The agreement of the nomogram with the criterion PIPR data was evaluated visually by plotting the differences between the spectral nomogram and criterion PIPR amplitude at each wavelength, and with Bland-Altman analysis (see Statistical Analysis). The PIPR data that were poorly described by the opn4 spectral nomogram were fitted with a rod photopigment (rhodopsin) CIE (1951) scotopic luminosity function [29–31] and also with a 10° cone photopic luminous efficiency (Vλ) function [32] by summing the L- and M-cone in a 1.625:1 ratio [33]. Since rods and ipRGCs, and to a lesser extent, cone photoreceptors contribute to the pupil light reflex [5,6,9,34–42], in cases where all of these three single photoreceptor nomograms poorly described the PIPR data, the data were fitted with the binary combination (opn4 + rhodopsin, opn4 + Vλ and rhodopsin + Vλ) or tertiary combination (opn4 + rhodopsin + Vλ) as defined by McDougal and Gamlin [34],

$$S(\lambda )={\left\{{\left\{m[S_{opn4}(\lambda )]\right\}}^{k2}+{\left[{\left({\left\{c\left[S_{cone}\left(\lambda \right)\right]\right\}}^{k1}+{\left\{r\left[S_{rod}\left(\lambda \right)\right]\right\}}^{k1}\right)}^{\frac{1}{k1}}\right]}^{k2}\right\}}^{1/k2}$$ (Eq 1)

where S(λ) is the combined spectral sensitivity of opn4 [S_opn4_(λ)] [5], the 10° photopic spectral luminous efficiency function [S_cone_(λ)] [32] and CIE scotopic luminosity function [S_rod_(λ)] with their relative contributions (m for opn4, c for cones, and r for rods). The model was fitted to the data by adjusting the free parameters to minimise the sum of squares of the differences between S(λ) and the criterion PIPR. The k_1_ and k_2_ parameters represent the Quick pooling model of visual sensitivity [43,44] for combining outer retinal (rhodopsin and Vλ) spectral sensitivities and both the outer retinal and inner retinal melanopsin sensitivities. These k_1_ and k_2_ parameters were systematically adjusted to optimise the S(λ) curve fit and then fixed at 1 and 11 for further curve fittings because these values provided the lowest sum of squared errors [34]. Fixing k_1_ and k_2_ limited the number of independently adjustable parameters involved in optimising the nomogram model fit.

### Statistical Analysis

Statistical data analysis was performed in GraphPad Prism (GraphPad Software, Inc., CA, USA). To determine the spectral nomogram that best described the PIPR, the deviation of the PIPR data from each nomogram was calculated. The agreement of either opn4, rhodopsin, Vλ or the combined opn4 + rhodopsin + Vλ nomograms with the criterion PIPR data was evaluated with Bland-Altman analysis, and the bias and 95% limits of agreement between the nomogram and criterion PIPR were reported [45]. The Bland-Altman analysis only reports bias and the limits of agreement can be estimated, however it does not provide a criterion for agreement; we are not aware of any literature that defines acceptable limits for estimating the photoreceptor spectral sensitivities.

To evaluate the differences in amplitude between 2, 3, 4, 5 and 6 s PIPR, a one-way repeated measures ANOVA (Geisser-Greenhouse correction, Tukey’s multiple comparisons, 95% confidence interval, p < 0.05) was performed. The CV of the PIPR amplitude was calculated as SD/Mean to determine the PIPR time metric with the lowest variability; intra-individual CV was based on at least two repeated measurements and inter-individual CV was based on the pupil data from 20 participants.

## Results

**Fig 3:**
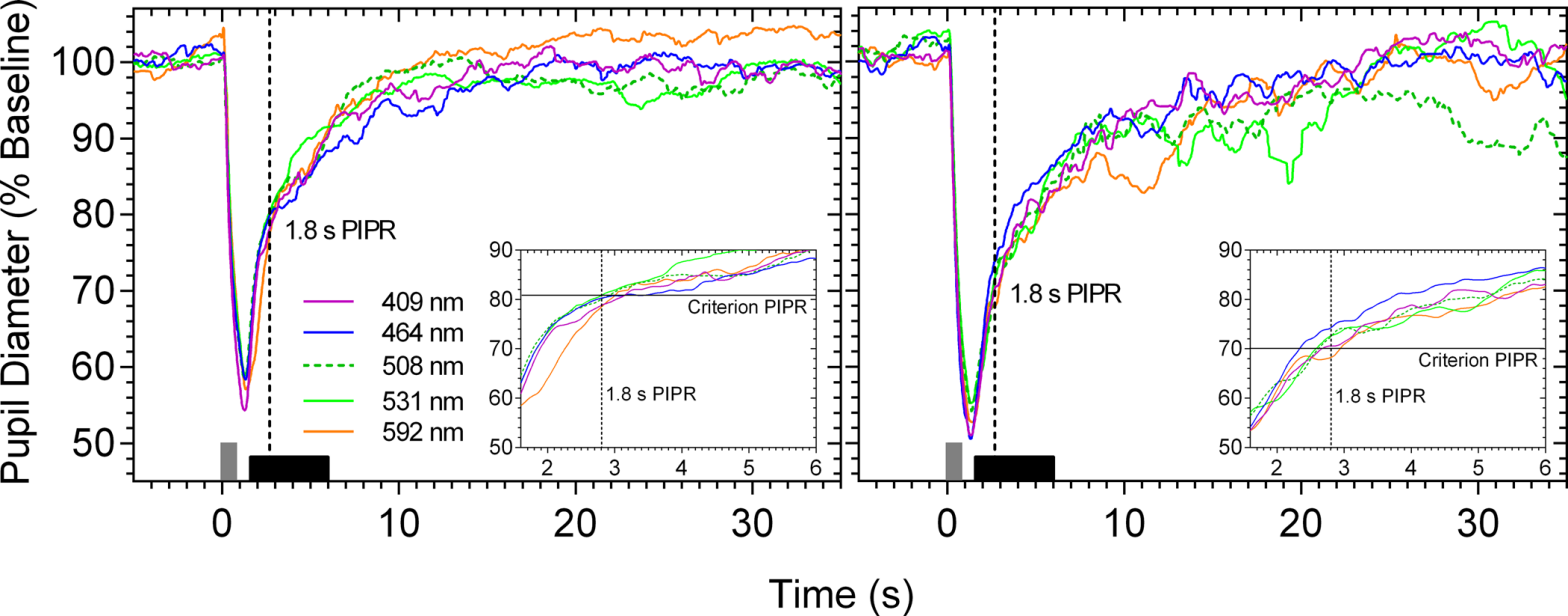
**Exemplar pupil light reflex traces of participant 32/F (left panel) and participant 31/M (right panel) in response to five test wavelengths to produce a criterion PIPR amplitude (% of baseline pupil diameter) at 1.8 s after light offset.** The vertical grey bar at 0 s indicates the 1 s stimulus pulse. The horizontal black bar along the abscissa indicates the post-stimulus period (0.6 to 5.0 s) where the PIPR spectral sensitivity was measured. The insets show a magnified view of the traces (0.6 to 5.0 s post-stimulus). The vertical dashed lines in all panels indicate the 1.8 s PIPR time and the horizontal solid lines in the insets indicate the criterion PIPR amplitude.

**Fig 4:**
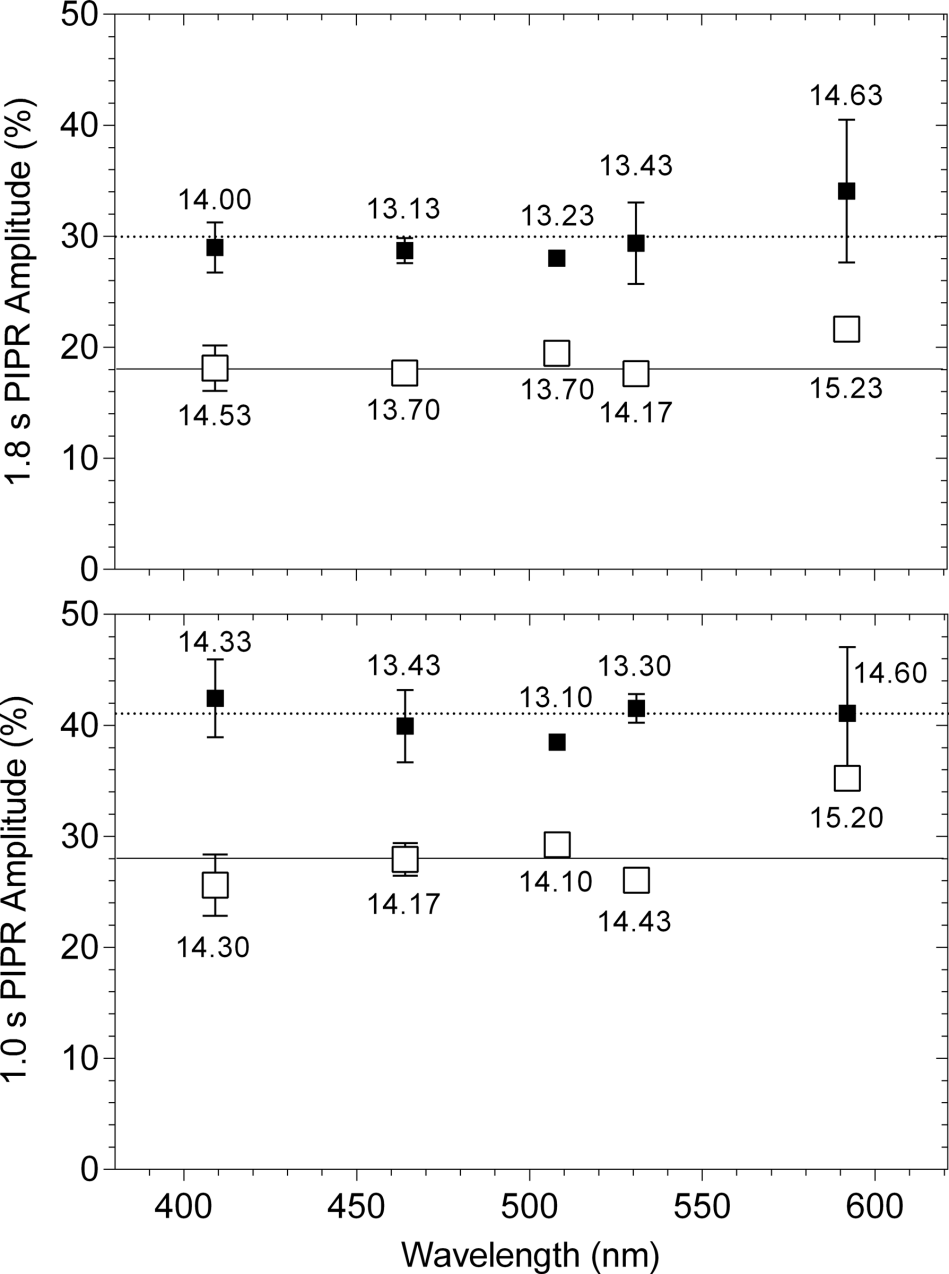
Difference (% of baseline pupil diameter) between the measured PIPR (symbols) and criterion PIPR (horizontal lines) for each primary light at 1.0 and 1.8 s after light offset. The unfilled and filled squares indicate the data (average ± SD) from participant 32/F and participant 31/M, respectively. The average retinal irradiance (log quanta.cm^-2^.s^-1^) required to produce the criterion PIPR is given for each wavelength.

When the criterion PIPR amplitude is measured for each primary light, the pupil light reflex traces overlap (dashed vertical line in Fig 3; criterion PIPR at 1.8 s). For all measured times after light offset at all wavelengths, the criterion PIPR amplitude could be achieved within ± 6.5% of the predefined criterion; the differences between the measured and criterion PIPR are shown in Fig 4 at two PIPR times, one that is described by the opn4 spectral nomogram (1.8 s) and the other that cannot be described by the opn4 nomogram (1.0 s). The difference between the measured and criterion PIPR was within the recommended acceptance limit of CV [46] and less than the reported CV of the 6 s PIPR amplitude (about 10%) [3].

**Fig 5:**
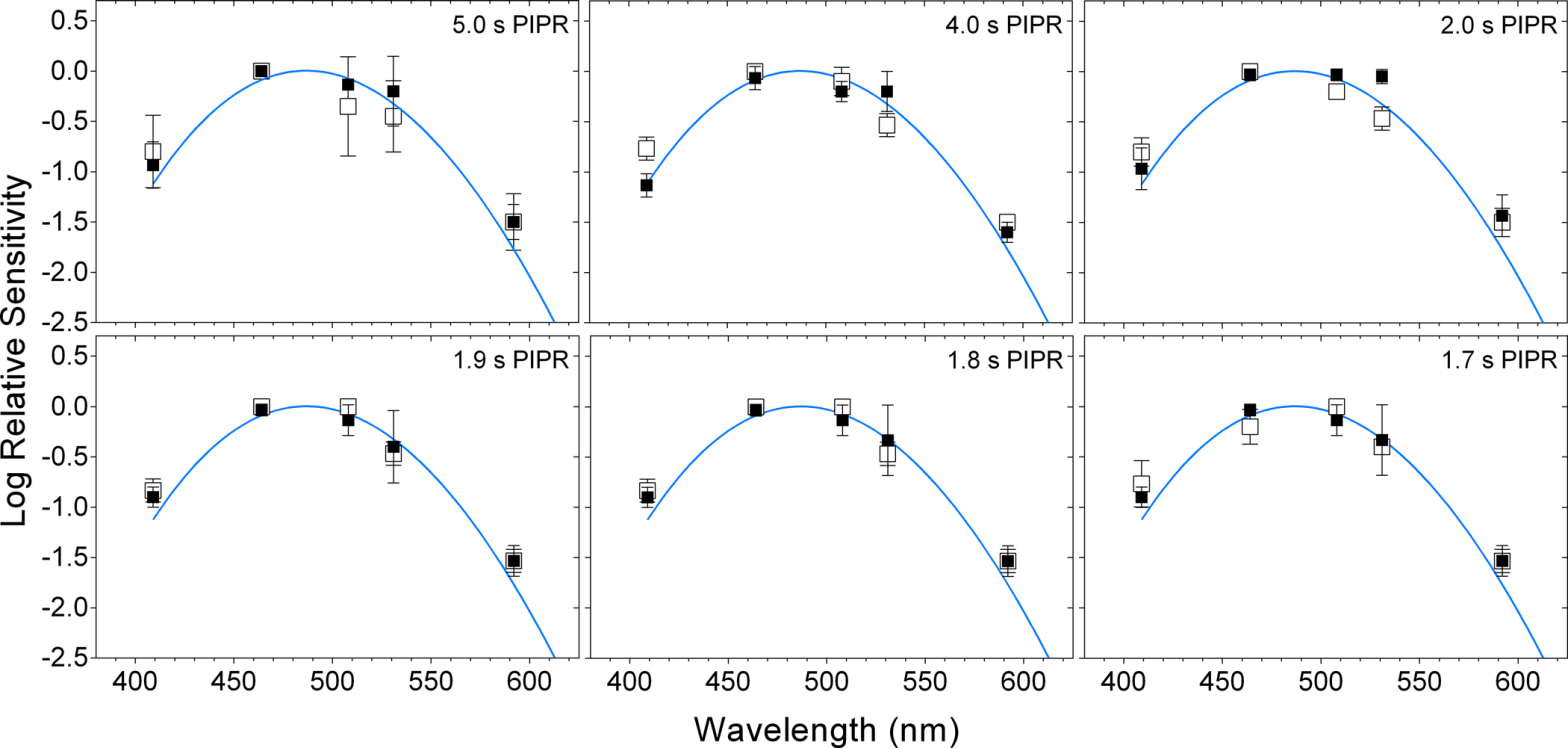
Spectral sensitivity of the post-illumination pupil response (PIPR) at 1.7 to 5.0 s after light offset. The unfilled and filled squares indicate the data (average ± SD) from participant 32/F and participant 31/M, respectively; the blue curves indicate the melanopsin (opn4) spectral sensitivity nomogram.

**Fig 6:**
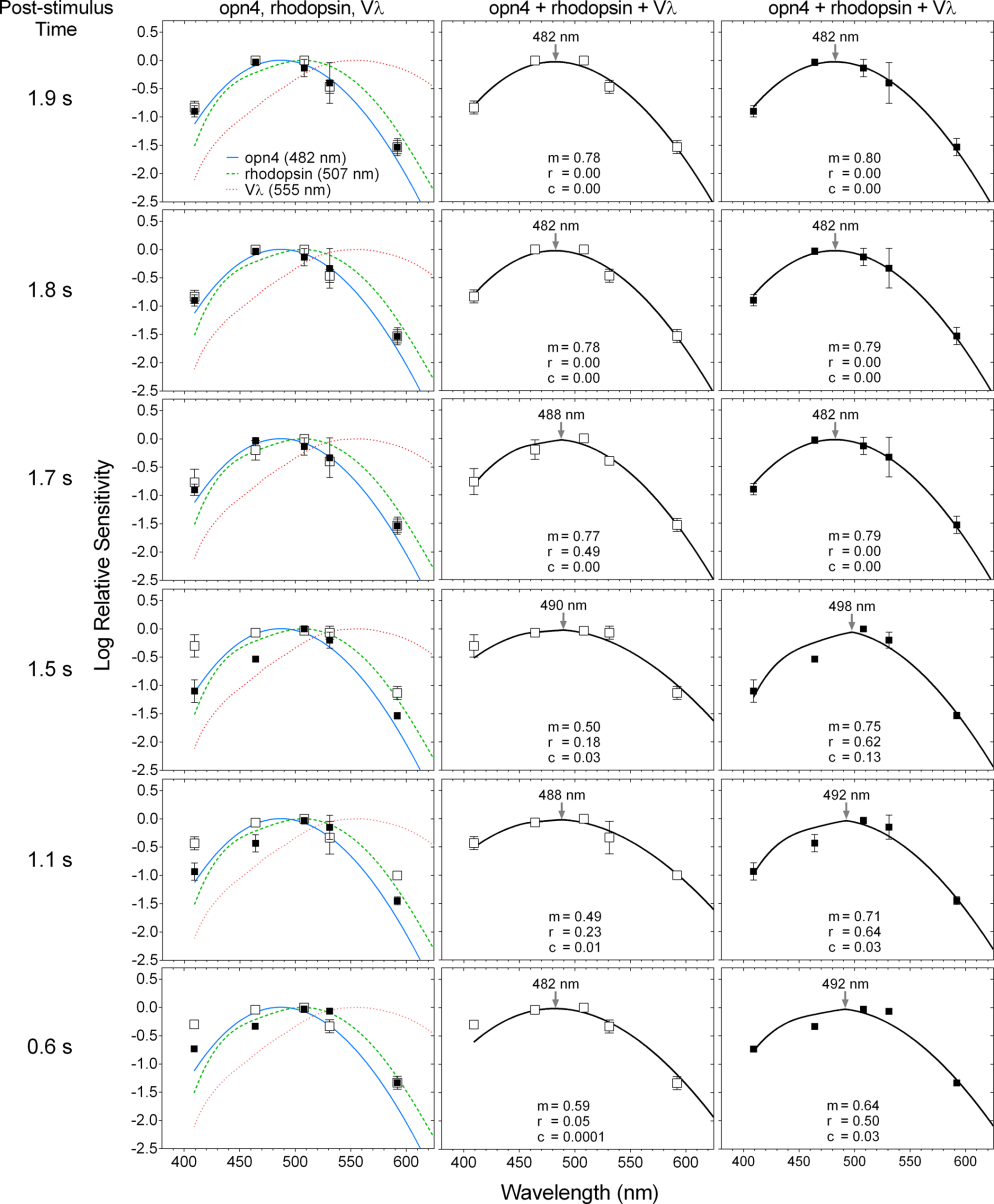
Spectral sensitivity of the post-illumination pupil response (PIPR) at 0.6 to 1.9 s after light offset. The unfilled and filled squares indicate the data (average ± SD) from participant 32/F and participant 31/M, respectively. The curve fitting with the opn4 + rhodopsin + Vλ nomogram is separately shown for each participant in the middle (32/F) and right (31/M) panels; m, r and c are relative contributions to the PIPR from opn4, rhodopsin and Vλ, respectively (Eq 1). The nomogram peaks are indicated by the arrows in the middle and right panels.

**Fig 7:**
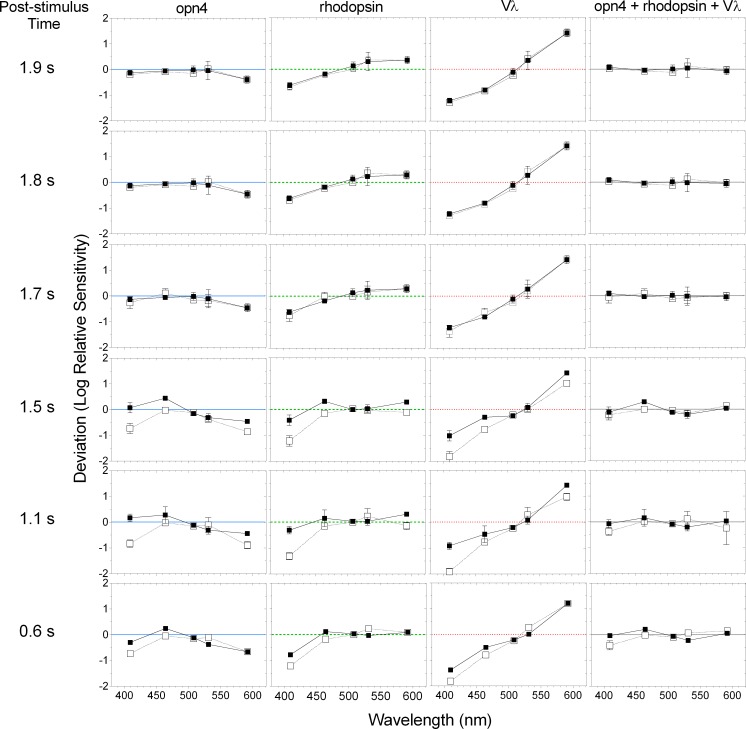
Deviation of the criterion PIPR data from the single opn4, rhodopsin and Vλ spectral sensitivity nomograms, and the combined opn4 + rhodopsin + Vλ nomogram at 0.6 to 1.9 s after light offset. The unfilled and filled squares indicate the data (average ± SD) from participant 32/F and participant 31/M, respectively; the horizontal lines indicate no deviation from the nomograms.

**Fig 8:**
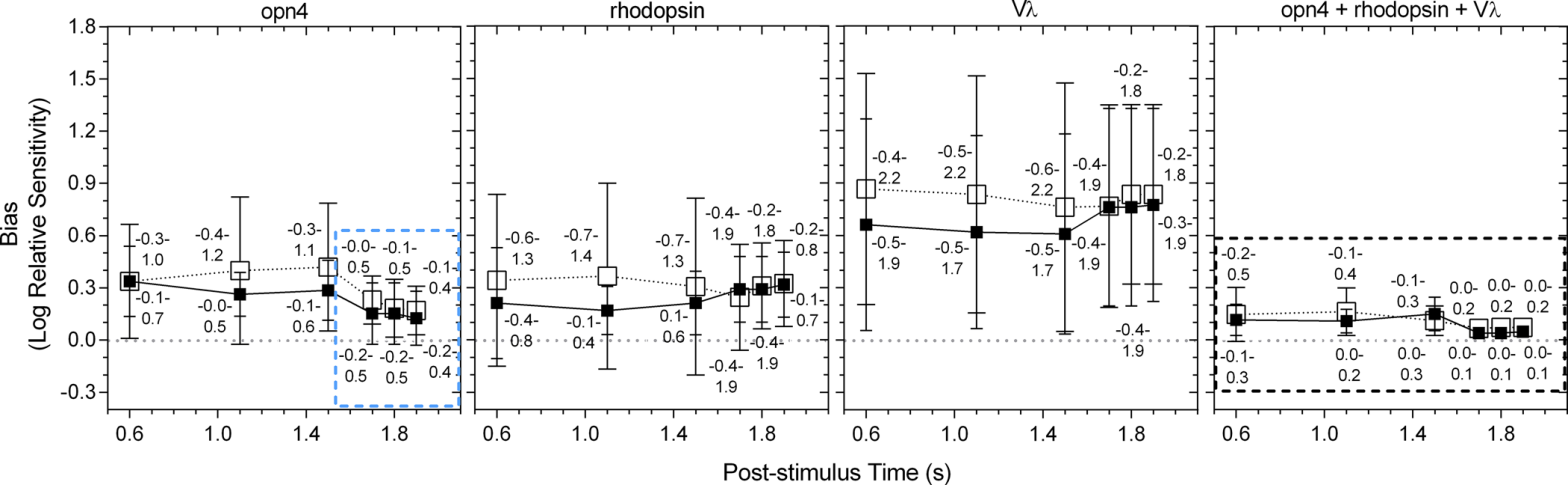
Bland-Altman analysis of the agreement between the criterion PIPR data and the single opn4, rhodopsin, Vλ spectral nomograms, and the combined opn4 + rhodopsin + Vλ nomogram at 0.6 to 1.9 s after light offset. The unfilled and filled squares indicate the data (average ± SD) from participant 32/F and participant 31/M, respectively. The numbers above and below the symbols indicate the 95% limits of agreement between the nomogram and PIPR for participant 32/F and participant 31/M, respectively. The horizontal dotted lines indicate zero bias; the dotted boxes highlight the nomograms providing the best fit.

For times > 1.7 s after light offset, a single photopigment nomogram with a peak sensitivity in the range of melanopsin (opn4, λ_max_ = 482 nm) (Fig 5; and Fig 6, upper-left two panels) described the criterion PIPR with the lowest deviation (Fig 7, upper two rows), least bias and narrowest 95% limits of agreement (Fig 8) compared to the other single nomograms. When the binary (opn4 + rhodopsin, opn4 + Vλ and rhodopsin + Vλ) and tertiary (opn4 + rhodopsin + Vλ) combination models were fitted to this data (> 1.7 s; Fig 6, upper two rows), the PIPR was completely described by the melanopsin nomogram (m) with zero weightings for the rhodopsin (r) and Vλ (c) contributions, and the model deviation decreased (Fig 7), the bias was lower and the limits of agreement were narrower than for the single nomograms (Fig 8). The quality of fit improved with the combined model because melanopsin contribution can be independently adjusted (parameter m, Eq 1) whereas the single nomogram model has no free parameters.

A transition between the opn4 nomogram and the combined model as the better description of the criterion PIPR became evident at 1.7 s post-stimulus, with participant 32/F requiring a 0.49 rhodopsin contribution in the tertiary model, although there was only a small difference in the deviation (Fig 7) and bias (Fig 8) between the 1.7 s and 1.8 s PIPR. For all post-stimulus times < 1.7 s, the single photopigment nomograms (Fig 6, left column) and binary combinations (not shown) had larger deviations (Fig 7, lower three rows), higher bias and wider 95% limits of agreement (Fig 8) than the tertiary combination of the spectral nomograms. The peak of the best-fitting tertiary model shifted away from the opn4 nomogram peak towards longer wavelengths (range: 482 to 498 nm; Fig 6, middle and right columns) with shorter post-stimulus times. The relative photoreceptor contributions to the PIPR at times < 1.7 s were dominated by melanopsin with major contributions from rods and minor contributions from cones, with some differences in the weights between the two participants, but not in their pattern. For all post-stimulus times, the Vλ nomogram showed the largest deviation, highest bias and widest 95% limits of agreement.

**Fig 9:**
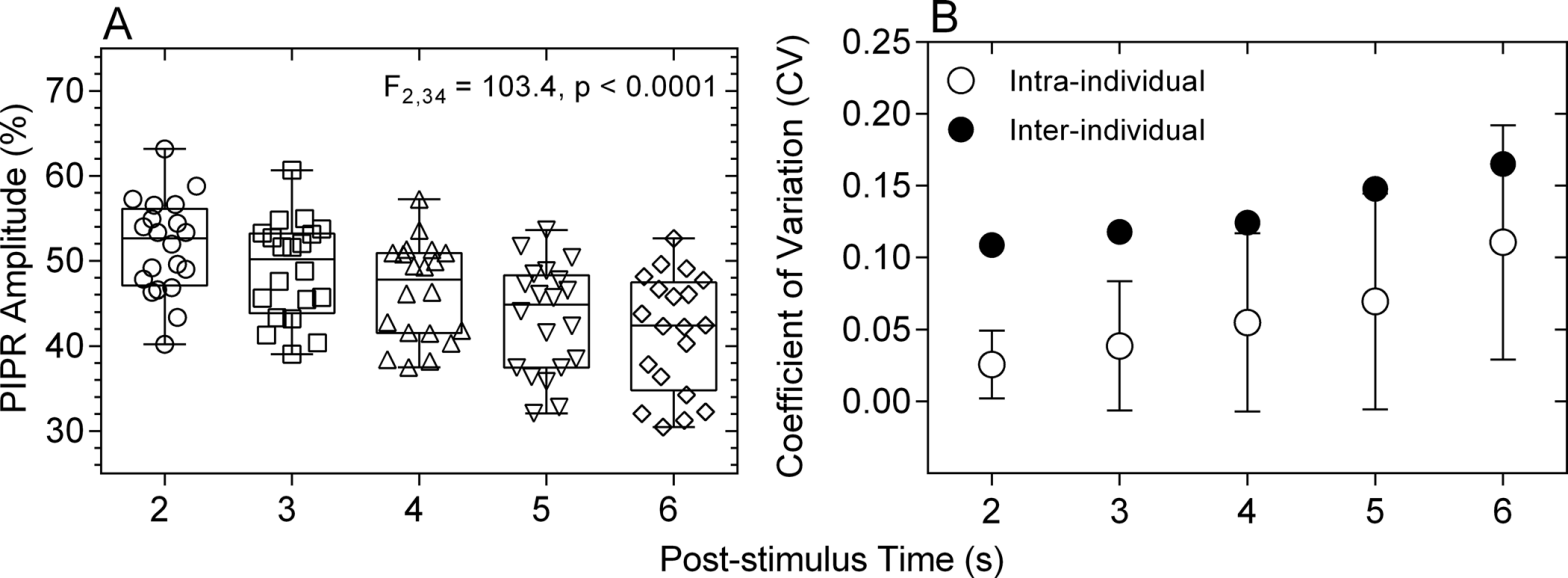
**Amplitudes (% of baseline pupil diameter) (Panel A) and intra- and inter-individual coefficients of variation (CV) (Panel B) of the 2, 3, 4, 5 and 6 s PIPR.** Data from a sample of 20 observers with normal ocular health, aged between 35 and 74 years. In panel A, smaller percentage baseline values on the ordinate indicate smaller PIPR amplitudes.

A PIPR metric with low variability is better able to differentiate the disease effects on ipRGCs from intra- and inter-individual PIPR variability. Having determined that the PIPR measured at > 1.7 s after light offset is entirely driven by melanopsin, we compared the PIPR amplitude and intra- and inter-individual coefficients of variation of the 2, 3, 4, 5 and 6 s PIPR in response to a 1 s, 464 nm, 15.5 log quanta.cm^-2^.s^-1^ pulse in a cohort of 20 participants. The PIPR amplitude differed significantly between the measured times (F_2,34_ = 103.4, p < 0.0001), with the mean PIPR amplitudes decreasing with increasing post-stimulus time (Fig 9A). The intra- and inter-individual CV increased with increasing post-stimulus time (Fig 9B).

## Discussion

The spectral sensitivity of the dark-adapted post-illumination pupil response (PIPR) to a 1 s stimulus pulse measured ≥ 1.7 s post-stimulus is entirely described by the melanopsin (opn4) photopigment spectral nomogram (peak at 482 nm) (Figs 5–8). For post-stimulus times < 1.7 s, the combination of three photoreceptor spectral nomograms (opn4 + rhodopsin + Vλ) best describes the spectral sensitivity of the PIPR (Figs 6–8) and the peak of this best fitting curve shifts to longer wavelengths (range: 482 to 498 nm) indicating major melanopsin and rhodopsin contributions and minor cone contributions.

The ipRGCs depolarise during light stimulation and repolarise slowly after light offset [16] to produce a sustained PIPR [1]. Therefore, melanopsin dominates all phases of the PIPR with major contributions in the early redilation phase between light offset and < 1.7 s post-stimulus and solely controls the PIPR at ≥ 1.7 s post-stimulus as observed in our data. Importantly, the PIPR < 1.7 s post-stimulus receives contributions from both outer and inner retinal photoreceptors with the peak of the best-fitting combination opn4 + rhodopsin + Vλ model shifting away from the melanopsin peak sensitivity (~482 nm) towards the rhodopsin peak spectral sensitivity (~507 nm), indicating major contributions from rhodopsin and melanopsin (Fig 6). Our finding of rhodopsin contributions to the early phase of the PIPR has not been reported previously in the human pupil data. Electrophysiology recordings in mice retinae demonstrate that recovery of the rod photoresponse after light offset ranges from ~0.5 s after single-photon stimulation [47] to ~5 s with a rod saturating stimulus (ranging in stimulus duration from 2 s to 100 s) [48]. In humans, the rod photoresponse recovery time to a 1 s, 3.3 log scot td.s (~12.4 log quanta.cm^-2^.s^-1^) light is ~9 s [49]. Hence, rod signalling is present shortly after light offset, which explains the notable rod contributions to the PIPR at < 1.7 s post-stimulus in our data (Fig 8), in line with evidence that rods contribute to the maintenance of the steady-state pupil constriction during continuous light stimulation, at least for durations < 10 s [34]. In comparison, cones have faster photoresponse kinetics [50] and make minor contributions to the steady-state pupil constriction [34] and the very early phase of the PIPR (< 1.7 s) (Figs 6 and 7). Based on our findings, we propose that measureable rhodopsin contributions to the PIPR recover and terminate by approximately 1.7 s after light offset (note that our lowest irradiance stimulus is ~7.0 log quanta.cm^-2^.s^-1^ above rod threshold) and melanopsin completely controls the PIPR thenceforward.

The post-stimulus time, when rhodopsin contribution to the PIPR in the opn4 + rhodopsin + Vλ model becomes zero and melanopsin entirely controls the model, differed by ~0.1 s between our two participants (1.8 s for participant 32/F and 1.7 s for participant 31/M; Fig 6). It is not possible to determine the origin of this variability with this small participant sample, but future analyses could consider the role of melanopsin gene polymorphisms that are known to alter the PIPR amplitude in healthy people [51] but it is unknown if these polymorphisms shift the opn4 peak spectral sensitivity. There is no evidence for rod gene polymorphisms in humans with normal visual function [52], whereas L- and M-cone gene polymorphisms can shift the peak of the Vλ nomogram [53,54] but any potential effect of this on our data would be negligible due to a minor or no cone contributions to the PIPR at all post-stimulus times (Fig 6).

The PIPR quantified with the 6 s metric has been shown to be effective in detecting melanopsin dysfunction in glaucoma [13,18,55,56], age-related macular degeneration [14] and ischemic optic neuropathy [15] in humans. However, the time after light offset when the PIPR should be measured for early disease diagnosis may differ with diseases depending on their pathophysiology. In early glaucoma, the 6 s PIPR provides the largest differentiation in melanopsin function from healthy eyes compared to other PIPR times [18], whereas in early AMD, the 12 s PIPR provides the largest differentiation [14]. The implication is that the selection of the post-stimulus time when the PIPR amplitude is measured should be determined based on the disease of interest. As such, further differentiation of PIPR metrics may be useful for clinical evaluation of disease effects on ipRGCs. Our finding that the PIPR measured at any time from 1.7 s after light offset is entirely melanopsin driven may allow for the optimal selection of a time that provides the largest differentiation between melanopsin function in eyes with and without disease. The secondary outcome of this study shows that the PIPR amplitudes decrease and the intra- and inter-individual coefficients of variation increase with increasing post-stimulus time from 2 s to 6 s (Fig 9) indicating that the PIPR measured closer to 2 s post-stimulus will have lower variability compared to the PIPR at longer post-stimulus times. A larger PIPR amplitude (Fig 9A) provides a larger dynamic range that will be more sensitive to melanopsin dysfunction in early disease stages and more robust to attenuation in stimulus retinal irradiance due to lenticular opacities in older persons. Based on our data, pupillometric paradigms used to measure ipRGC function do not solely need to depend on short wavelength lights near the peak melanopsin sensitivity, but any wavelength can be used to produce a similar PIPR amplitude by adjusting the stimulus irradiance, including longer wavelength lights (e.g., amber appearing lights) that are more robust to lens attenuation.

In conclusion, rhodopsin and melanopsin contribute to the early phase of the dark-adapted PIPR (< ~1.7 s post-stimulus), in line with the electrophysiological observations of rod signalling shortly after the cessation of a light stimulus, and melanopsin solely drives the PIPR at longer post-stimulus times.
